# Discovery of mutated oncodriver genes associated with glioblastoma originated from stem cells of subventricular zone through whole exome sequence profile analysis, and drug repurposing

**DOI:** 10.1016/j.heliyon.2025.e42052

**Published:** 2025-01-16

**Authors:** Arnob Sarker, Burhan Uddin, Reaz Ahmmed, Sabkat Mahmud, Alvira Ajadee, Md. Al Amin Pappu, Md. Abdul Aziz, Md. Nurul Haque Mollah

**Affiliations:** a Bioinformatics Lab (Dry), Department of Statistics, University of Rajshahi, Rajshahi, 6205, Bangladesh; b Department of Biochemistry and Molecular Biology, University of Rajshahi, Rajshahi, 6205, Bangladesh

**Keywords:** Glioblastoma (GBM), Whole exome Sequence (WES) profile, Somatic mutation, Oncodriver genes, Drug repurposing, Integrated bioinformatics analysis

## Abstract

Glioblastoma (GBM) is one of the most aggressive cancers due to its high mortality rate in spite of intensive treatment. It may be happened because of drug resistance against their typical receptors, since these receptor genes are often mutated by environmental stress. So identifying mutated oncodriver genes which could be used as potential drug target is essential in order to develop effective new therapeutic drugs as well as better prognosis for GBM patients. In this study, we analyzed whole exome sequencing (WES) profiles of NCBI database on GBM and matched-normal (control) samples originated from astrocyte like neural stem cells (NSC) of subventricular zone (SVZ) to explore GBM-causing mutated oncodriver genes, since SVZ is considered as the origin of GBM development. We detected 16 mutated oncodriver genes. Then, filtering by differential co-expression analysis based on independent RNA-Seq profiles of CGGA database revealed 10 genes as dysregulated oncodriver genes. Following that, 3 significantly overexpressed oncodriver genes (MTCH2, VWF, and WDR89) were identified as potential drug targets. Then molecular mechanisms of GBM development were investigated by these three overexpressed driver genes through gene ontology (GO), KEGG-pathways, Gene regulatory network (GRN) and mutation analysis. Finally, overexpressed oncodriver genes guided top-ranked six drug agents (Irinotecan, Imatinib, etoposide, pazopanib, trametinib and cabozanitinib) were recommended against GBM through molecular docking study. Most of our findings received support by the literature review also. Therefore, the findings of this study might carry potential values to the wet-lab researchers for further investigation in terms of diagnosis and therapies of GBM.

## 1 Introduction

**Fig. 1:**
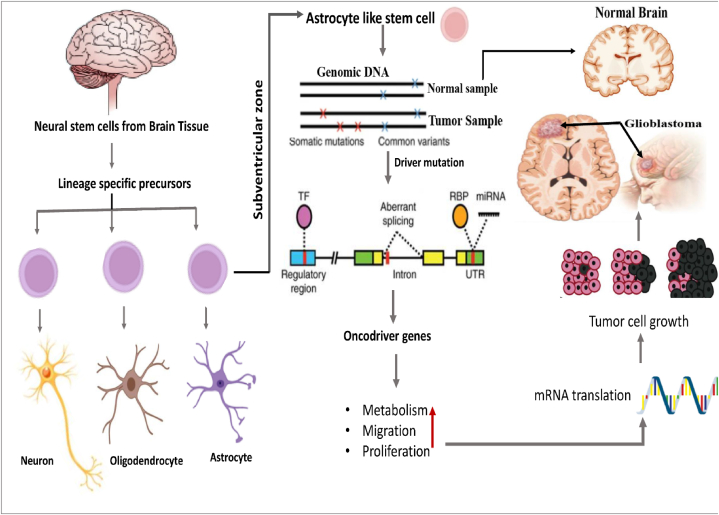
Schematic Diagram of the development of GBM from astrocyte-like stem cells originating in the subventricular zone.

Glioblastoma (GBM) is the most frequent aggressive primary brain tumor [1]. It is well-known for its high mortality rate in spite of intensive treatment like temozolomide-based chemoradiation or maximal safe resection [2,3]. It accounts for approximately 57 % of all gliomas and 48 % of all primary malignant central nervous system (CNS) tumors [4]. Generally GBM develops as de novo primary tumor or some time from lower-grade astrocytoma as secondary tumor through continuous progression [5]. The prognosis remains poor due to its rapid progression, with survival rates of 42.4 % at 6 months and 17.7 % at 1 year [6]. Tumor development typically occurs when cells accumulate somatic mutations and epigenetic modifications. Somatic mutations are classified into driver and passenger mutations based on their impact on cancer initiation and progression. The majority of genetic mutations in cancer patients are occurred as passenger mutations, which do not significantly influence cancer development [7]. In contrast, driver gene's mutation play a crucial role in promoting cancer initiation and progression by leading to abnormal cell proliferation (Fig. 1) [8].

Cancer stem-like cells, particularly neural stem cells (NSCs) in the subventricular zone (SVZ) has been identified as the origin of GBM [[9], [10], [11], [12]]. NSCs are multipotent and self-renewing, with the capacity to differentiate into neurons, astrocytes, and oligodendrocytes, the primary cell types of the brain [13,14]. In addition, partially differentiated oligodendrocyte precursor cells (OPCs) and astrocyte precursor cells, may contribute to tumor genesis. A recent study has investigated the mutational status of typical GBM causing driver genes based on Whole Exome Sequence (WES) profiles generated from the astrocyte-like NSCs and found low level of driver mutations in case of non-tumor SVZ. These low level driver mutations migrate from SVZ and lead to high-grade gliomas in distinct brain regions [15]. However, after the tumor forms in a specific region, the mutational landscape may shift, potentially leading to the emergence of new driver genes [16,17]. Furthermore, detecting rare driver mutations is particularly challenging due to their low frequency of occurrence, as well as their specificity to particular regions or tissues [18]. Additionally, the overexpression of oncodriver genes in cancer makes them as key tumor-associated antigens that can trigger an antitumor immune response [19]. Moreover, their strong association with cancer makes them promising drug targets [20]. Thus, disclosing new oncodriver genes those are involved in the development of GBM is essential to uncover the underlying causes, progression and candidate drug agents.

In this study, we aimed to identify some new oncodriver genes based on gain of function mutation through the analysis of WES profiles in GBM generated from NSCs niche belonging to the SVZ, because gain of function mutation are mostly responsible for cancer migration and invasion [16,21,22]. Different bioinformatics tools including Genome Analysis Toolkit (GATK) were used to detect somatic mutated genes from WES profiles. After that, oncodriver genes were identified from the somatic mutated genes-set by using OncodriveCLUST [23]. In order to identify co-differentially expressed oncodriver genes between GBM and control samples, RNA-Seq profiles were analyzed by using the Weighted Gene Co-Expression Network Analysis (WGCNA) approach [24]. Then we identified significantly overexpressed oncodriver genes through the combination of independent TCGA and GTEx databases [25]. We also disclosed their mechanism of disease development by investigating gene ontology (GO) terms, KEGG-pathways and gene regulatory networks (GRN). Finally, an attempt was made to explore repurposable drug molecules against GBM by molecular docking analysis. Thus, this study may provide valuable insights into the pathogenetic mechanisms of GBM, offering potential targets for therapeutic intervention and contributing to the development of novel drugs.

## 2 Materials and methods

**Fig. 2:**
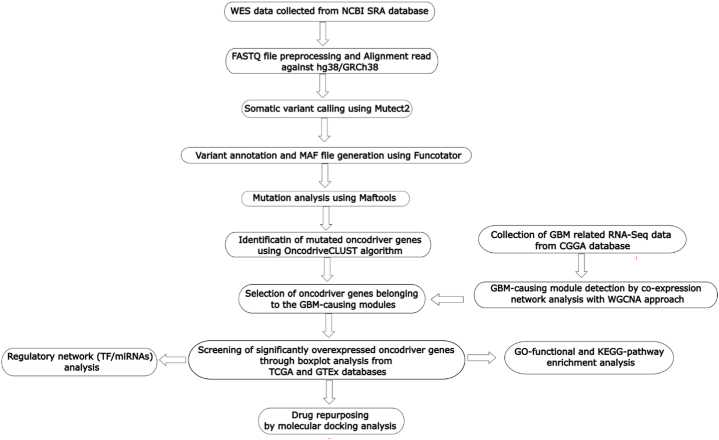
The workflow of this study.

To explore GBM-causing oncodriver genes, we analyzed whole exome sequencing (WES) profile dataset by using sratoolkit [26], FastQC [27], GATK [28], maftools [29], ‘WGCNA’ R-package [24], GEPIA2 web tool [25] etc. In order to disclose GBM development and progression through oncodriver genes, we investigated gene ontology (GO), KEGG-pathways and regulatory networks by using Enrichr [30], NetworkAnalyst server [31], Cytoscape [32]. Then oncodriver gene guided drug molecules were explored by using PyMOL [33], AutoDock Vina [34]. The detail descriptions of materials and methods were given sections 2.1-2.7 and the workflow was displayed in Fig. 2.

### 2.1 Data source and description

To explore oncodriver genes for GBM, we collected tumor and matched normal Whole Exome Sequencing (WES) profile dataset from NCBI SRA database with accession ID SRP145073 [15]. To identify co-differentially expressed oncodriver genes, RNA-Seq data was collected from CGGA database containing 62 case and 20 control samples with their metadata [35]. Additionally, brain cancer-related candidate drug molecules were collected by reviewing two articles [36,37]. The detailed information of the datasets are given in supplementary documents (Table S1, S2 and S3).

### 2.2 Disclosing oncodriver genes through WES profiles analysis

Initially, sratoolkit.3.1.0 [26] was used to obtain and dump FASTQ files. FastQC (v .0.11.8) [27] tool were used to check the quality of the FASTQ files. Next, BWA-MEM (v 0.7.18 -r1243) was utilized to align the reads to the human reference genome, hg38/GRCh38 [38]. Marking duplicate reads and base quality score recalibration (BQSR) were performed by genome analysis toolkit (GATK version 4.3) [28]. Mutect2 tumor with matched normal mode was employed to detect the somatic variant from the tumor samples using default parameters to generate Variant Calling Format (VCF) files [39]. Variants were filtered using FilterMutectCalls in accordance with the GATK website's recommended practices [40]. Subsequently, Funcotator was used to annotate the variants and create a Mutation Annotation Format (MAF) file from the VCF data. Lastly, the further analysis was carried out using the R package maftools (v 2.22.0) [29]. ‘plotmafSummary’ and ‘oncoplot’ functions were utilized to interpret the overall mutational status. The function “tcgaCompare” was used to compare tumor mutational burden against 33 TGCA cohorts. The “lollipopPlot” function of maftools was used to show the distribution of mutation in the unregulated oncodriver genes [29].

The “oncodrive” function was utilized to identify potential driver genes with false discovery rate (FDR) less than 0.1 and keeping other parameters default. The “oncodrive” function, based on the OncodriveCLUST algorithm, identifies potential driver genes by leveraging the fact that most cancer-causing gene variants are concentrated at specific loci (hotspots) [23]. That is why at first, the mutational positions of candidate amino acids which exceeds above threshold are compiled for every genes. Next, the candidates whose locations fall within five amino acid distances from one another are clustered together and cluster score is calculated. It does so by performing five key steps. These steps include I. Mutations Retrieval, ii. Identification of Cluster Seeds, iii. Clusters Formation, IV. Completion of Clusters and finally V. Scoring the Clusters shown in the equation:

$$Clusteringscore\;=\sum_{i}\frac{fractionMutations}{{\left(\sqrt[{\;}]{2}\right)}^{distance}}$$

Here, the variable i represents the protein positions within the cluster. The term fractionMutations refers to the percentage of mutations at that specific position relative to the total mutations observed in the protein across all samples and distance indicates the number of amino acids between positions i and the position in the cluster that exhibits the highest number of mutations. The “somaticInteractions” function was utilized to examine the co-occurrence or mutually exclusive pair of frequently oncodriver genes with P-value less than 0.1. This function conducts pairwise Fisher's exact tests to identify significant gene pairs.

### 2.3 Screening of oncodriver genes by their dysregulating analysis through WGCNA approach

Oncodriver genes are usually dysregulated [41,42]. To eliminate oncodriver genes by testing their dysregulating patterns between GBM and control samples, we considered RNA-Seq profile dataset (Case = 62, control = 20) from CGGA database. At first, co-differentially expressed oncodriver genes between GBM and control samples were identified by using the Weighted Gene Co-Expression Network Analysis (WGCNA) approach with ‘WGCNA’ R-package (v 1.72–5) [24]. The "hclust" function was utilized to illustrate the presence of any outlier samples present. Then, data was normalized using variance stabilizing transformation [43]. Genes, which had no expression in more than 75 % samples were excluded. Those surviving genes then assembled into an adjacency matrix by Pearson correlation with setting the power to soft threshold β. Following this, a topological overlap matrix (TOM) and dissimilarity TOM (1-TOM) was constructed. Moreover, the "cutreeDynamic" function was used to identify modules [44]. Module eigengene (ME) was then calculated to create a new merged module by merging modules with high similarity scores. Close neighboring MEs were merged to create new MEs. After that, to construct module-trait relationship ‘cor’ function was used to compute Pearson correlation between MEs and the phenotypes (conditions) of the samples. A module of genes were eliminated if module-trait relationship is insignificant (*p*-value >0.05).

### 2.4 Identification of overexpressed oncodriver genes and the impact of their mutations

As upregulated (overexpressed) oncodriver genes are usually considered as drug targets [45,46], we explored upregulated oncodriver genes by differential expression pattern analysis between GBM and control samples through box plots based on combined GTEx and TCGA databases in the GEPIA2 web tool [25]. Then we used ‘PolyPhen-2’ [47] and ‘SNPs&GO’ [48] to predict the functional impact of the identified mutations found in the significantly overexpressed oncodriver genes to better understand its role in GBM.

### 2.5 Detection of regulatory factors of overexpressed oncodriver genes

We investigated how transcription factors (TFs) and microRNAs (miRNAs) regulate the oncodriver genes at both the transcriptional and post-transcriptional stages by analyzing their regulatory networks. The JASPAR database [49] was used to identify the main TFs and the TarBase database [50] was used to explore the main miRNAs. The NetworkAnalyst server [31] was used to produce the networks. We used Cytoscape to visualize their interaction networks [32].

### 2.6 Functional enrichment analysis of overexpressed oncodriver genes

The Gene Ontology (GO) project is a bioinformatics tool that uses domain-specific ontologies to provide a complete source of functional data on gene products and descriptions of activities [51]. To investigate the Gene Ontology and KEGG pathway of oncodriver genes, we considered Enrichr [30] database with *P*-value of 0.05 was chosen as threshold.

### 2.7 Drug repurposing guided by overexpressed oncodriver genes

We considered overexpressed oncodriver genes (proteins) as drug target (receptor). In order to investigate the interaction between the overexpressed oncodriver protein-drug complexes, molecular docking was performed. At first, the sequences of the proteins (receptor) were retrieved from Uniprot [52] and underwent homology modelling by SWISS-MODEL [53] using template to obtain the 3D structures (Table S5). Then, all the mutations, which were obtained from WES data analysis, were induced at the exact position of the particular proteins using PyMOL (v 3.0.3) [54]. Detail information are given in (Table S6). The 3D structures of the candidate drugs (ligand) were retrieved from the PubChem database [55]. All receptors and ligand's structure underwent energy minimization and the binding affinity scores (BAS) (kcal/mol) were then calculated by molecular docking using AutoDock Vina (v 1.5.7) [34]. Docking was performed in two scenarios: (i) between non-mutated (original) proteins and drugs, serving as the control, and (ii) between mutated proteins and drugs, serving as the case. This approaches aimed to evaluate whether mutations in protein level caused by alteration in the sequences of genes, affect drug binding. After docking, changes in interaction types and interacting atoms between proteins and drug molecules were visualized using Discovery Studio [56] to assess their role in altering binding affinity [57,58].

## 3 Results

### 3.1 Disclosing oncodriver genes through WES profiles analysis

**Fig. 3:**
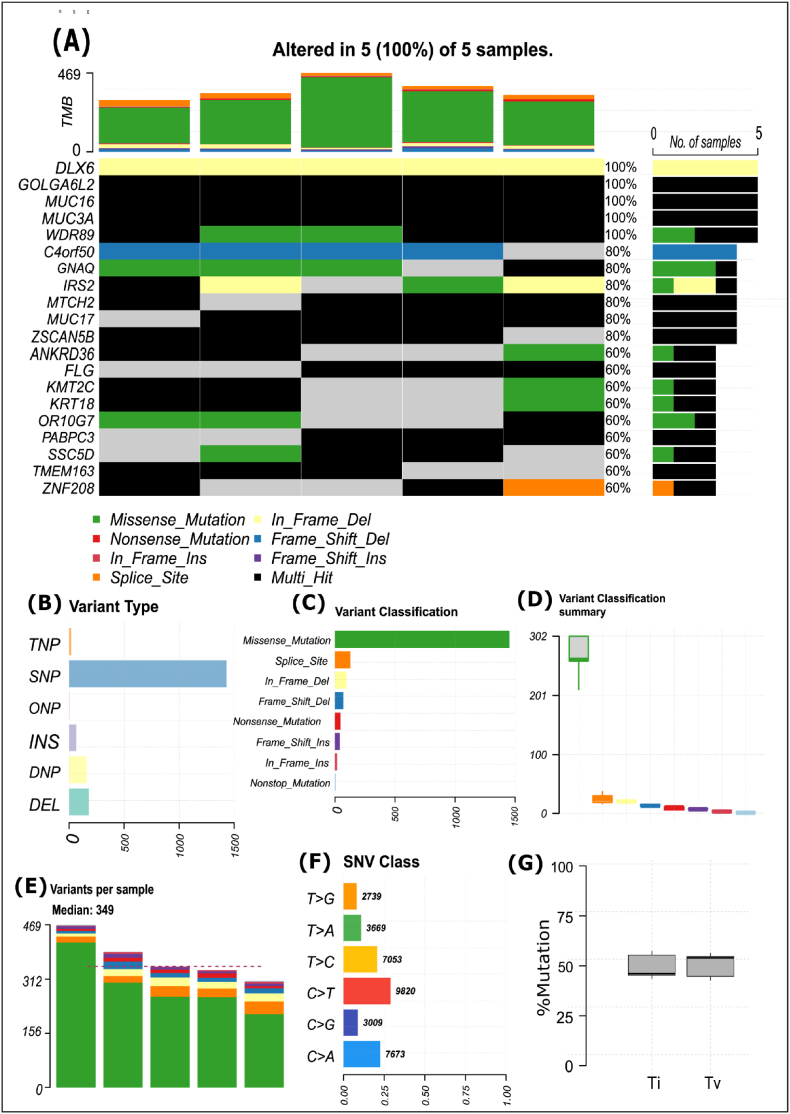
Analysis of somatic mutational landscape of glioblastoma arises from subventricular zone. **(A)** Brick plot of somatic mutations in frequently and significantly mutated genes. Genes are represented in horizontal lines and the samples are represented in vertical lines while various types of mutations are indicated by different colors. **(B)** Variant type shows the highest number is SNP (single nucleotide polymorphism) **(C**–**D)** Variant classification reveals that missense mutations are the most (E) Variant per sample shows median variant is 349 **(F)** SNV (single nucleotide variants) class shows the highest number of alteration is C > T **(G)** Transitions (Ti) vs. transversions (Tv) plot shows the median of Tv is slightly higher than Ti.

All the FASTQ files successfully passed the quality control checks. After analyzing the MAF files, which were generated through GATK best practice pipeline, 33963 Single nucleotide variation (SNV) were detected within 5 samples (Fig. 3F). Total 11023 somatic mutated genes were identified with at least one mutation. Among the SNV classes, C > T transitions (Ti) accounted for the largest proportion, comprising 28.9 % of the total but overall transversion's (Tv) median was found to be greater than Ti (Fig. 3G). Additionally, the median number of variants identified in the five samples was 349, with the highest count being 469 and the lowest count being 310 (Fig. 3E). Moreover, the SNV classes was the most dominant variant type among others (Fig. 3B). Furthermore, variant classification showed that, missense mutation ranked first among all (Fig. 3C) as well as there were notable variations gap between missense and other various variant classes (Fig. 3D). Top 20 mutated genes across 5 samples were, DLX6 (100 %), GOLGA6L2 (100 %), MUC16 (100 %), MUC3A (100 %), WDR89 (100 %), C4orf50 (80 %), GNAQ (80 %), IRS2 (80 %), MTCH2 (80 %), MUC17 (80 %), ZSCAN5B (80 %), ANKRD36 (60 %), FLG (860 %), KMT2C (60 %), KRT18 (60 %), OR10G7 (60 %), PABPC3 (60 %), SSC5D (60 %), TMEM163 (60 %), ZNF208 (60 %) (Fig. 3A). From (Fig. S1), it can also be seen that the TMB found in our analysis was much more higher compared to other TCGA cohorts.

**Fig. 4:**
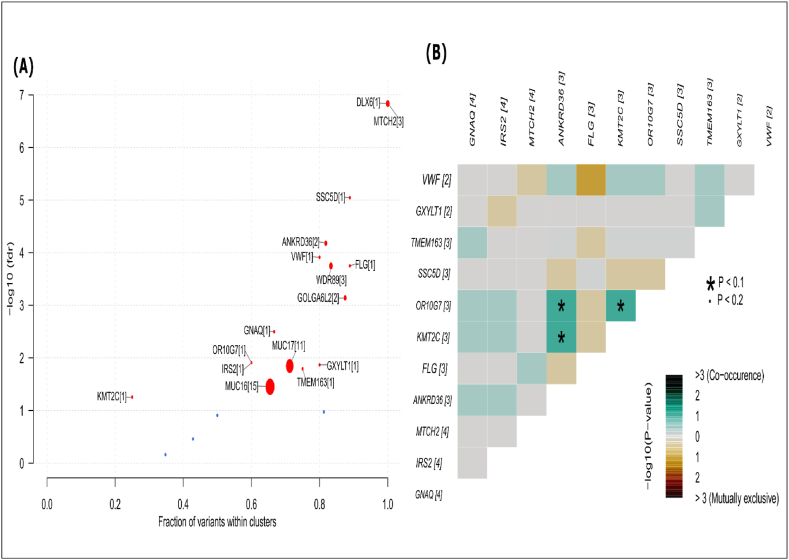
Oncodriver gene identification from somatic mutation. **(A)** GBM-associated driver genes, identified through oncodrive analysis with an FDR <0.1. These genes are presented along with the number of mutational clusters, which are indicated in brackets. **(B)** Mutually co-occurring oncodriver gene pairs depicted in a triangular matrix.

Several oncodriver genes were identified from the mutated genes based on their clustering characteristics around mutational hotspots namely, DLX6, MTCH2, SSC5D, ANKRD36, VWF, FLG, WDR89, GOLGA6L2, GNAQ, MUC17, OR10G7, IRS2, GXYLT1, TMEM163, MUC16 and KMT2C (Fig. 4A, Fig. S2). The analysis of somatic interactions among the driver genes revealed three distinct instances (ANKRD36 and OR10G7, ANKRD36 and KMT2C and ORG10G7 and KMT2C) of mutual co-occurrence events (Fig. 4B).

### 3.2 Screening of oncodriver genes by dysregulating analysis through WGCNA approach

**Fig. 5:**
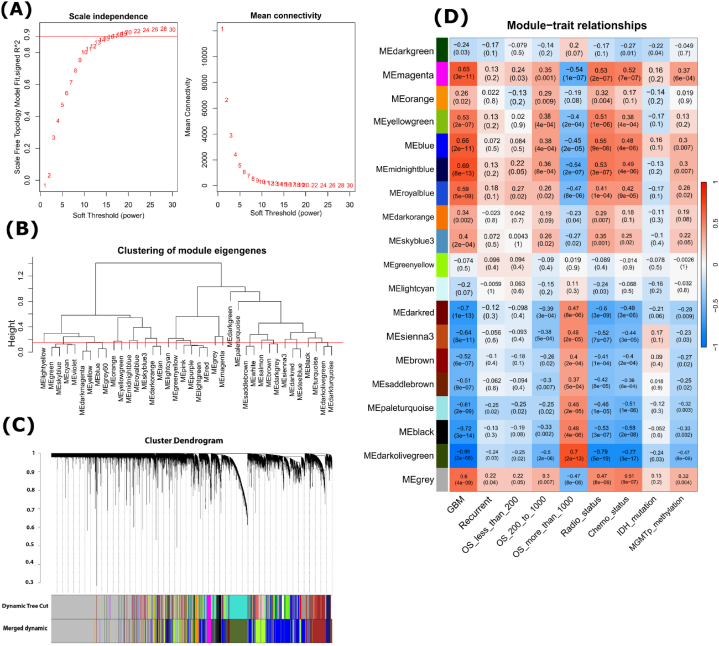
Weighted Gene Co-expression Network Analysis (WGCNA) of GBM. **(A)** Selection of soft threshold for matrix construction. **(B)** Clustering of module eigengene for merging neighbor modules. **(C)** Cluster Dendrogram of merged and unmerged modules. The various modules are indicated by the colors of the horizontal bar. Some color variation in merged dynamic compared to dynamic tree cut, depicts the merging of several close modules. **(D)** Module-trait relationship. The color shift from orange to blue indicating a transition from positive to negative correlation with their corresponding P-value.

Co-expression network analysis was done using RNA-Seq data to identify significant modules and their association with different GBM traits. No outlier sample was observed (Fig. S3). Total 24256 genes were obtained from 55521 genes after excluding the low expressed genes. To make sure that, the gene expression network follow the power law distribution and maintain the biological network, a soft threshold β = 16 was chosen for at R^2^ value of 0.9 (Fig. 5A). Then, hierarchical clustering was performed to identify modules with minimum module size 30. To merge closely neighboring modules, cut height of Module Eigengene (ME) was set to 0.15 (Fig. 5B). (Fig. 5C) displays all gene modules both before and after merging [59]. To find out the most significant modules associated with GBM and where the oncodriver genes were co-expressed within those modules, modules-traits relation was constructed between the MEs and phenotypes (Fig. 5D). We figured out that, 8 oncodriver genes were co-expressed in grey, 3 in greenyellow, and 2 in blue modules (Table S4). Thus, grey is the most representative module. Further analysis revealed that, grey module has a significant positive correlation with GBM, recurrence of GBM, overall survival (OS) less than 200 and 200–1000 days, radio therapy, chemotherapy and MGMT promoter methylation (*p*-value <0.05). On the other hand, grey module showed negative correlation with overall survival more than 1000 days (*p*-value <0.05), which may have occurred because most of the GBM patients shifted to the ‘death-group’ (Table S2). Blue module also followed almost same correlation pattern as grey module with different traits. However, greenyellow module with 3 oncodriver genes showed insignificant correlation with GBM and other traits (*p*-value >0.05). That's why we excluded the oncodriver genes found in this module for further analysis.

### 3.3 Identification of overexpressed oncodriver genes and the impact of their mutations

**Fig. 6:**
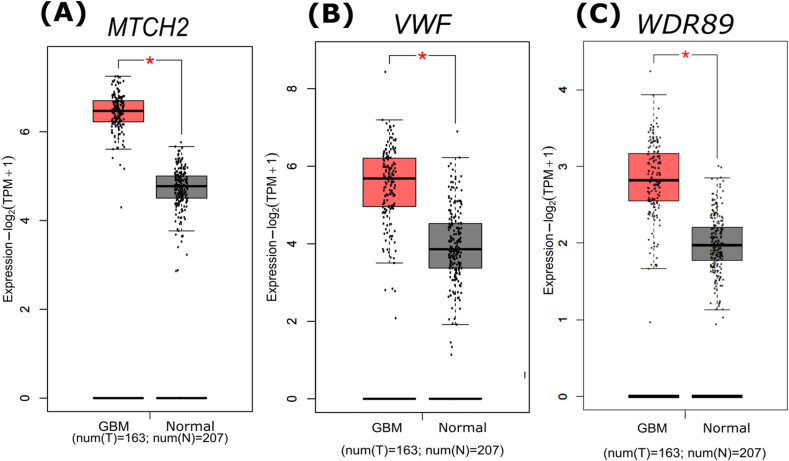
(A–C) Box plot to visualize significantly overexpressed oncodriver genes. GBM samples are represented in red and normal samples are represented in black.

Differential expression status of the oncodriver genes found in grey and blue modules were investigated through using GTEx and TCGA that collectively contains 163 GBM and 207 control. We found that, out of 10 oncodriver genes MTCH2, VWF, and WDR89 were significantly upregulated (Fig. 6). We considered these 3 upregulated oncodriver genes as drug target. Moreover, multiple in-silico tools PolyPhen-2’ [47] and ‘SNPs&GO’ [48] predicted that most of the identified variants in these 3 genes are disease causing and damaging with high probability.

**Fig. 7:**
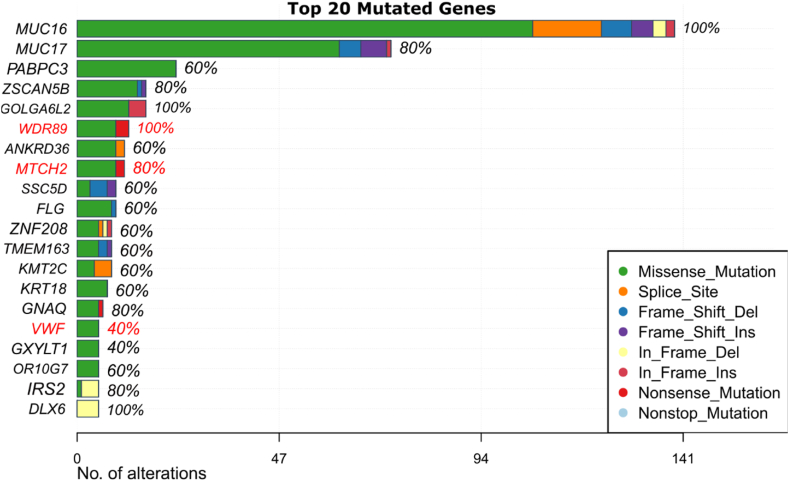
The analysis of the top 20 mutated genes reveals key patterns in mutation frequency and mutation types. The x-axis represents the number of alterations across all analyzed samples, providing a quantitative measure of mutation burden for each gene. Genes highlighted in red are the overexpressed oncodriver genes found in our analysis.

Further somatic mutational status of the 20 mutated genes were depicted by bar plot where these 3 upregulated oncodriver genes are highlighted in red color (Fig. 7). These genes (WDR89, MTCH2 and VWF) are ranked 6th, 8th, and 16th respectively among the top 20 mutated genes. Notably, MUC16, GOLGA6L2, WDR89, and DLX6 exhibit the highest mutation frequency at 100 % across the samples analyzed. Among the three oncodriver genes, WDR89 and MTCH2 exhibit both missense and nonsense mutations, with missense mutations being more common, while VWF contains only missense mutations.

### 3.4 Detection of regulatory factors of overexpressed oncodriver genes

**Fig. 8:**
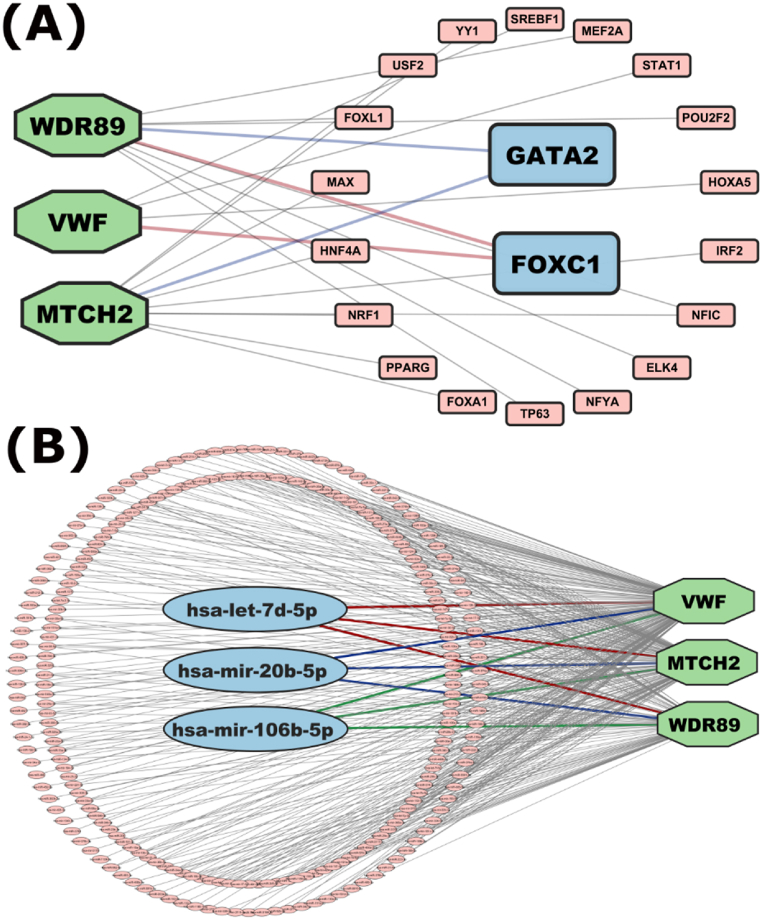
Regulatory network of the over-expressed oncodriver genes marked as light-green colored hexagonal shape. **(A)** Network of TFs-overexpressed oncodriver genes interaction. TFs are marked as rectangular shape. Top ranked two TFs are marked as sky-blue color. **(B)** The miRNA-overexpressed oncodriver genes interaction network. miRNAs are marked as oval shape. Top ranked three miRNAs are marked as sky-blue color.

The transcription factors (TFs) and micro RNAs (miRNAs) networks were used to examine the regulators of overexpressed oncodriver genes. We chose the top two TFs (GATA2, FOXC1) according to two topological measures, betweenness and degree with cutoff of 105.57 and 3 respectively as they play most prominent role in transcriptional level of the Oncodriver genes (Fig. 8A). By employing the exact topological measures method, we chose the top three miRNAs (hsa-mir-20b-5p, hsa-let-7d-5p, hsa-mir-103a-3p) with betweenness and degree cutoff of 288 and 5 respectively (Fig. 8B).

### 3.5 Functional enrichment analysis of overexpressed oncodriver genes

For oncodriver genes, we carried out GO and KEGG pathway analysis. Here we took into account the most important GO terms from each cellular component (CC), biological process (BP), molecular function (MF), and KEGG pathways with P-value <0.05 (Table 1).

**Table 1:** The top significantly (p-value<0.05) enriched GO functional and KEGG pathways by oncodriver gene set.

	Biological Process			
GO ID	Description	p-value	Adjusted P-value	Annotated genes
GO:0007599	Hemostasis	0.000899	0.008068	VWF
GO:0010635	Regulation of Mitochondrial Fusion	0.001649	0.008068	MTCH2

**Cellular Component**				
GO ID	**Description**	**p-value**	**Adjusted P-value**	**Annotated genes**

GO:0062023	Collagen-Containing Extracellular Matrix	0.011654	0.044304	VWF
GO:0031093	Platelet Alpha Granule Lumen	0.009867	0.044304	VWF
GO:0005741	Mitochondrial Outer Membrane	0.022480	0.049001	MTCH2

**KEGG Pathways**				
NO	**description**	**p-value**	**Adjusted P-value**	**Annotated genes**

1	ECM-receptor interaction	0.013143	0.045866	VWF
2	Platelet activation	0.018486	0.045866	VWF
3	Focal adhesion	0.029849	0.045866	VWF

### 3.6 Drug repurposing guided by overexpressed oncodriver genes

**Fig. 9:**
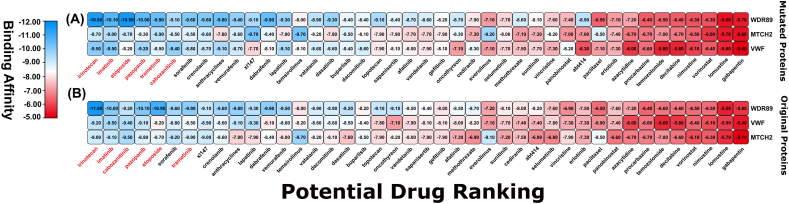
Binding affinity score of **(A)** Mutated protein-drug and **(B)** Original protein-drug complexes. The color transition from red to blue indicates a shift from lower to higher binding affinity. A higher negative value indicates better binding.

The 3 overexpressed oncodriver proteins (MTCH2, VWF and WDR89) and their corresponding mutated one were subjected for molecular docking analysis. The binding affinity scores (BAS) were calculated between these proteins and candidate drugs. There was a significant change of BAS between non-mutated and mutated protein-ligand complexes. WDR89 was the protein which ranked top among all in both mutated and non-mutated form. VWF was in second highest rank among the non-mutated complexes based on overall BAS, but its position dropped to third after mutation induction. Conversely, MTCH2 ranked third under original conditions but rose to the second position after mutation had been incorporated (Fig. 9). Moreover, we found that the six compounds namely Irinotecan, Imatinib, etoposide, pazopanib, trametinib and cabozanitinib produced an average binding affinity score (BAS) < −9.0 kcal/mol. Thus, we proposed these six drugs as the top-ranked candidates for inhibiting the proposed oncodriver genes.

We further investigated the interaction types and interacting atoms between the protein-drug complexes. We figured out that, after incorporating mutations, the BAS of WDR89 & irinotecan complex was increased (from −11.60 to −10.80) where some new interactions are included like A:TYR282:N -:LEGAND:O, A:CYS328:SG -:LEGAND:O and some were omitted like A:PHE327:N -:LEGAND:O, A:SER326:CA -:LEGAND:O etc. along with the changes of interaction types. On the other hand, the BAS of WDR89 & imatinib complex was decreased (from −10 to −10.10) after incorporating mutations, where some new interactions are included like LEGAND:H - A:CYS221:SG, A:ARG77:NH2 -:LEGAND etc. and some were omitted like A:CYS221:SG -:LEGAND (Table 2).

**Table 2:** Interaction between proteins (main and mutated) and top 2 receptor compounds (irinotecan and imatinib). The third column displays the changes of interaction position before and after mutation incorporation along with the type of interaction. The fourth column displays the interacting atoms and molecules between amino acids of the receptor proteins and ligands. The newly included and omitted interactions are highlighted in bold.

Protein and Ligand complex	Binding Affinity (kCal/mol)	Target-Ligand Interaction	Interaction between Amino Acids and drugs molecules
**WDR89 (Main)** **& irinotecan**	−11.60		**A:PHE327:N -:LEGAND:O** **A:SER326:CA -:LEGAND:O** :LEGAND:C - A:PHE327:O **:LEGAND:C - A:ARG325:O** A:ARG77:NH2 -:LEGAND **A:TYR233:OH -:LEGAND** **A:PRO180 -:LEGAND** **:LEGAND:C - A:LEU281** **:LEGAND:C - A:CYS221** **A:PHE327 -:LEGAND:C** **:LEGAND - A:CYS221**
**WDR89 (Mutation)** **& irinotecan**	−10.80		**A:TYR282:N -:LEGAND:O** **A:CYS328:SG -:LEGAND:O** **:LEGAND:H - A:TYR233:OH** **:LEGAND:C - A:ILE29:O** :LEGAND:C - A:PHE327:O **:LEGAND:C - A:SER220:O** **:LEGAND:C - A:THR174:O** A:ARG77:NH2 -:LEGAND **A:CYS221:SG -:LEGAND** **A:ILE119 -:LEGAND** **A:ARG372 -:LEGAND**
**WDR89 (Main)** **& imatinib**	−10.00		A:ARG77:NE -:LEGAND:N A:ARG77:NH2 -:LEGAND:O A:ARG177:NH2 -:LEGAND:O :LEGAND:H - A:TYR233:OH :LEGAND:C - A:SER220:O :LEGAND:C - A:LEU277:O A:ASP122:OD2 -:LEGAND **A:CYS221:SG -:LEGAND** A:PHE327 -:LEGAND :LEGAND:C - A:LEU281 :LEGAND - A:PRO180
**WDR89 (Mutation)** **& imatinib**	−10.10		A:ARG77:NE -:LEGAND:N A:ARG77:NH2 -:LEGAND:O A:ARG177:NH2 -:LEGAND:O **:LEGAND:H - A:CYS221:SG** :LEGAND:H - A:TYR233:OH :LEGAND:C - A:SER220:O :LEGAND:C - A:LEU277:O **A:ARG77:NH2 -:LEGAND** A:ASP122:OD2 -:LEGAND A:PHE327 -:LEGAND :LEGAND:C - A:LEU281 **:LEGAND - A:CYS221** :LEGAND - A:PRO180

## 4 Discussion

Despite the advancement in cancer research, Glioblastoma (GBM) is still a highly aggressive brain tumor with poor prognosis, due to the heterogeneity and frequent genetic mutation to the patients. After diagnosis, the survival rates of GBM patients are 42.4 % at 6 months and 17.7 % at 1 year [6]. Therefore, in-depth molecular research is required for better diagnosis and therapies. In this study, we considered Whole Exome Sequence (WES) profile dataset generated from the astrocyte-like neural stem cells (NSCs) within the subventricular zone (SVZ) to discover some novel GBM-causing oncodriver genes as therapeutic targets. At first, 11023 somatic mutated genes with at least one mutation were detected from WES profiles by using GATK tools, where most of the variants found in the ‘missense mutation’ class (Fig. 3C & D). In most cases, missense mutations influence the structure and function of proteins or enzymes by altering the genetic information [60]. It was found that missense mutations are predominant and also contribute to the pathogenesis of GBM [60,61]. Among the variant types, single nucleotide polymorphism (SNP) was predominant and C > T alteration was most common followed by C > A (Fig. 3B & F). SNP can promote and regulate cancer in different ways, e.g. affecting TATA box, altering transcription factor binding site, changing coding regions etc. [63]. Multi studies have identified the role of SNPs in the diagnosis and prognosis of GBM [64,65] along with a higher frequency of C > T alteration [62,66]. We found that the median value of Tumor Mutation Burden (TMB) is 8/mb, which is the second highest in the TCGA cohorts (Fig. S1). It should be noted here that more mutations in a tumor lead to a higher TMB, which indicates better response to immunotherapy [67].

Then top-ranked GBM-causing 16 oncodriver genes (DLX6, MTCH2, SSC5D, ANKRD36, VWF, FLG, WDR89, GOLGA6L2, GNAQ, MUC17, OR10G7, IRS2, GXYLT1, TMEM163, MUC16 and KMT2C) were detected from the somatic mutated gene-set (Fig. 4A) by using the OncodriveClust algorithm. Furthermore, gene-gene interaction analysis revealed the 3 mutually co-occurrence patterns of the oncodriver genes as ANKRD36 and OR10G7, ANKRD36 and KMT2C, and ORG10G7 and KMT2C (Fig. 4B). Similarly, mutually co-occurrence patterns of mutated genes were also investigated in other cancer studies [68]. Then we identified co-differentially expressed oncodriver genes by using WGCNA approach. We observed that oncodriver genes belong to three modules, where two modules (grey and blue) containing 10 genes (MUC16, WDR89, MTCH2, SSC5D, FLG, TMEM163, GNAQ, IRS2, DLX6, and VWF) were statistically significant (Fig. 5D & S4). They showed positive correlation with radio status, chemo status, MGMT promoter methylation and negative correlation with longer overall survival indicate their strong association with GBM. Expression analysis of oncodriver genes from TCGA database showed three genes (VWF, MTCH2, and WDR89) are significantly overexpressed (Fig. 6A–C) that may be considered as potential drug targets [20]. After that tentative mechanisms of GBM development and progression by this three oncodriver genes were discussed as follows.

Among the suggested oncodriver genes Von Willebrand factor (VWF), typically expressed in endothelial cells and megakaryocytes, and has been linked in cancer metastasis. Research demonstrated the de novo expression of VWF in glioma, suggesting a potential new avenue for understanding its involvement in brain tumors [69]. We have also find three mutations at p.(R1342C), p.(L1503P) and p.(S1506L) which all are missense mutation. It is very common in various disease that VWF exhibits missense mutations, accounting for 75 % of all mutations [70]. Additionally, we discovered that FOXC1 is a transcription factor for VWF which was found significant in promoting tumor growth and angiogenesis [71]. The results of the enrichment analysis revealed a strong correlation between the biological process of hemostasis and cellular components such the platelet alpha granule lumen and collagen-containing extracellular matrix. It has been found that VWF is present in one of the platelet secretory granules, known as α-granules [72] which is typically associated with platelet activation pathway [73] and the negative modulation of angiogenesis [74]. Platelet activation ultimately lead to primary hemostasis. However, alterations in the genetic regulation of VWF expression can disrupt this process, leading to an increased risk of thrombosis [75]. Evidence said Thromboembolic diseases is common in high-grade glioma [76].

One more overexpressed oncodriver gene suggested is, Mitochondrial carrier homolog 2 (MTCH2) is a protein located on the mitochondrial outer membrane that interacts with truncated BH3-interacting domain death agonist (tBID) to regulate cell apoptosis [77]. Functional enrichment analysis revealed its association with Regulation of mitochondrial fusion. Abnormal functioning of mitochondrial outer membrane protein has been found in the development and progression of various types of cancer [78]. Moreover, knocking down MTCH2 in various glioma cell lines (A172 and U87MG) resulted in reduced migration/invasion. In addition, we found one nonsense mutation at p.(R77∗) and three missense mutations at p.(C79H), p.(F237S), and p.(V246A) in MTCH2. A substitution of A > G in the MTCH2 gene at Chr11: 47647265 was identified as potentially deleterious in highly aggressive glioblastoma [79]. Besides, mutations in mtDNA, which contribute to the regulation of mitochondrial fusion, can also lead to cancer [80]. Furthermore, we found miRNA (hsa-mir-20b-5p) as a key regulator for MTCH2. A existing evidence showed that association between miRNA and MTCH2 is responsible for the development of breast cancer [81] (Fig. 6, Fig.S2, Fig.7 & Table 1).

Finally, the oncodriver gene suggested by us is WDR89, belongs to one of the biggest families of eukaryotic WD40-repeat (WDR) proteins. Nevertheless, the role of WDR proteins in brain development is not well understood. So far, brain disorders have been linked to mutations in 27 out of the 286 WDR genes that have been identified in both the human and mouse genomes (9.4 %) [82]. We identified four missense mutation at p.(K57R), p.(P70S), p.(R103S), p.(G112D) along with two nonsense mutation at p.(R64∗), p.(R100∗) in WDR89. Studies also suggested mutations in WDR89 gene which is linked to neuroanatomical abnormalities [82]. Additionally, WDR89 has been observed to be overexpressed in adamantinomatous craniopharyngioma (ACP), the most common primary brain tumor in the sellar region among children [83]. Moreover, we identified GATA2 as a transcription factor for WDR89. A study also demonstrated that GATA2 overexpression led to increased proliferation of SW1783 glioma cells, whereas GATA2 knockdown notably inhibited the proliferation of U87 glioma cells [84].

To further investigate the ability of our upregulated oncodriver genes (receptor protein) to be potential drug target, we conducted molecular docking studies and identified top six candidate drug (Irinotecan, Imatinib, etoposide, pazopanib, trametinib and cabozantinib), which demonstrated strong binding affinities. This result indicates oncodriver genes potential to be a suitable drug target (Fig. 9) [[85], [86], [87]]. These six drug molecules are FDA approved with Drug Bank (DB) accession ID DB00762, DB00619, DB00773, DB06589, DB08911and DB08875. Among the suggested drugs, Irinotecan block topoisomerase I enzyme, which is essential for DNA replication, transcription, and repair. The referenced article suggests that Irinotecan therapy has been shown significant anticancer activity against GBM [88]. Additionally, the 6-month overall survival rate was notably better with the combination of bevacizumab and irinotecan, showing a rate of 77 % compared to 60 % with temozolomide in GBM patient [89]. Imatinib is a potent inhibitor that targets a range of tyrosine kinases, including KIT, PDGFR, and Bcr-Abl, thereby disrupting their signaling pathways and inhibiting cell proliferation, as demonstrated in various preclinical studies [90]. Imatinib demonstrated tumor-inhibiting properties in primary cell cultures of human GBM [91]. Further interaction analysis between WDR89 and top 2 drugs (Irinotecan and Imatinib) revealed that, the interaction site and type of the protein was changed after incorporating mutations (Table 2). The role of interaction site and type has been a significant factor in the field of drug design and discovery [92,93].

Though this study provides a brief overview of discovering oncodriver genes which could be a potential drug targets, wet lab validation is necessary for the deep understanding of their role in GBM diagnosis, prognosis and therapies. Additionally, *in vivo* and *in vitro* validation of the drug molecules are needed to check their ability as potential inhibitor.

## 5 Conclusions

This study uncovered GBM-causing 16 mutated oncodriver genes from whole exome sequencing (WES) profiles generated from astrocyte like neural stem cells (NSC) of subventricular zone (SVZ). These genes were partitioned in to two significantly differential co-expression groups containing 2 genes in one module (FLG, GNAQ) and 8 genes in another module (MUC16, WDR89, MTCH2, SSC5D, TMEM163, IRS2, DLX6, and VWF). Then upregulated differential expression patterns of those co-expressed genes were separated by TCGA database and found 3 significantly upregulated/overexpressed oncodriver genes (MTCH2, VWF, and WDR89) as drug targets. The oncodriver genes regulatory network analysis revealed two TFs (FOXC1 and GATA2) and three miRNAs (hsa-mir-20b-5p, hsa-let-7d-5p, hsa-mir-103a-3p) as the transcriptional and post-transcriptional regulators. The driver genes-set enrichment analysis with GO-terms and KEGG pathways revealed some crucial biological process (Hemostasis, Regulation of Mitochondrial Fusion), cellular component (Collagen-Containing Extracellular Matrix, Platelet Alpha Granule Lumen) and pathways (Platelet activation, ECM-receptor interaction). Moreover, this study identified the top six drug candidates (Irinotecan, Imatinib, etoposide, pazopanib, trametinib and cabozanitinib) for targeting GBM, based on a molecular docking analysis of overexpressed oncodriver genes. Therefore, these findings not only enhance our understanding of GBM pathogenesis but also leading the way towards more effective therapeutic strategies.

## CRediT authorship contribution statement

**Arnob Sarker:** Writing – original draft, Visualization, Validation, Software, Methodology, Formal analysis, Data curation, Conceptualization. **Burhan Uddin:** Writing – original draft, Visualization, Validation, Software, Formal analysis, Data curation. **Reaz Ahmmed:** Visualization, Validation. **Sabkat Mahmud:** Software, Formal analysis. **Alvira Ajadee:** Software, Formal analysis. **Md. Al Amin Pappu:** Software, Formal analysis. **Md. Abdul Aziz:** Writing – review & editing, Project administration, Funding acquisition, Conceptualization. **Md. Nurul Haque Mollah:** Writing – review & editing, Supervision, Project administration, Funding acquisition, Conceptualization.

## Ethics approval and consent to participate

Not applicable, since the datasets analyzed in this study were downloaded from NCBI and CGGA databases that are freely available.

## Consent for publication

Not applicable.

## Data availability

The datasets analyzed in this study were downloaded from NCBI and CGGA databases by using the following link:

https://www.ncbi.nlm.nih.gov/Traces/study/?acc=SRP145073&o=acc_s%3Aa

http://www.cgga.org.cn/download.jsp.

## Declaration of competing interest

The authors declare that they have no known competing financial interests or personal relationships that could have appeared to influence the work reported in this paper.
