# Supplementary material for: Discovery of mutated oncodriver genes associated with glioblastoma originated from stem cells of subventricular zone through whole exome sequence profile analysis, and drug repurposing
Source: Heliyon. 2025 Jan 16;11(2):e42052. doi: 10.1016/j.heliyon.2025.e42052 (PMC11791140; doi:10.1016/j.heliyon.2025.e42052)
Supplement: Multimedia component 1 [file mmc1.docx]

**Supplementary files**

**Discovery of Mutated Oncodriver Genes associated with Glioblastoma Originated from Stem Cells of Subventricular Zone through the Whole Exome Sequence Profile Analysis, and Drug Repurposing**

Arnob Sarker^1^, Burhan Uddin^1,2^, Reaz Ahmmed^1,2^, Md. Sabkat Mahmud^1^, Alvira Ajadee^1^, Md. Al Amin Pappu^1^, Md. Abdul Aziz^2*^, and Md. Nurul Haque Mollah^1^*

^1^Bioinformatics Lab (Dry), Department of Statistics, University of Rajshahi, Rajshahi 6205, Bangladesh

^2^Department of Biochemistry and Molecular Biology, University of Rajshahi, Rajshahi 6205, Bangladesh

***Corresponding Author**(s) E-mail: Md. Abdul Aziz ([maazizbio@ru.ac.bd](mailto:maazizbio@ru.ac.bd)) and Md. Nurul Haque Mollah ([mollah.stat.bio@ru.ac.bd](mailto:mollah.stat.bio@ru.ac.bd))

**Co-Author’s Emails:** [arnobsarker05@gmail.com](mailto:arnobsarker05@gmail.com) (Arnob Sarker), [burhan8.314@gmail.com](mailto:burhan8.314@gmail.com) (Burhan Uddin), [reazahmmed147@gmail.com](mailto:reazahmmed147@gmail.com) (Reaz Ahmmed), [sabkatmahmud@gmail.com](mailto:sabkatmahmud@gmail.com) (Md. Sabkat Mahmud), [alviraajadee@gmail.com](mailto:alviraajadee@gmail.com) (Alvira Ajadee), [alamin.stat.bd@gmail.com](mailto:alamin.stat.bd@gmail.com) (Md. Al Amin Pappu)

## **Supplementary Table**

## Table S1: Detail information of WES data.

| ID | CASE | CONTROL | SAMPLE_ID |
| --- | --- | --- | --- |
| 1 | SRR7138406 | SRR7138414 | 160 |
| 2 | SRR7138415 | SRR7138417 | 396 |
| 3 | SRR7138418 | SRR7138421 | 276 |
| 4 | SRR7138422 | SRR7138424 | 245 |
| 5 | SRR7138439 | SRR7138441 | 499 |

## Table S2: Detail information of RNA-Seq data used for co-expression network analysis. 1 represents presence and 0 represent absence of the respective traits.

| CGGAID | GBM | Rec-  urrent | OS  Less  Than  200 | OS  200  To  1000 | OS  More  Than  1000 | Radio  status | Chemo  status | IDH  Mu-  tation | MGMTp  Methy-  lation |
| --- | --- | --- | --- | --- | --- | --- | --- | --- | --- |
| CGGA1036 | 1 | 0 | 0 | 1 | 0 | 1 | 1 | 0 | 1 |
| CGGA1041 | 1 | 0 | 0 | 0 | 1 | 1 | 1 | 0 | 0 |
| CGGA1075 | 1 | 0 | 0 | 1 | 0 | 1 | 1 | 0 | 0 |
| CGGA1103 | 1 | 0 | 0 | 1 | 0 | 1 | 1 | 1 | 1 |
| CGGA1106 | 1 | 0 | 0 | 1 | 0 | 1 | 0 | 0 | 1 |
| CGGA1130 | 1 | 1 | 0 | 1 | 0 | 1 | 0 | 1 | 1 |
| CGGA1134 | 1 | 0 | 1 | 0 | 0 | 0 | 1 | 0 | 1 |
| CGGA1135 | 1 | 0 | 0 | 0 | 1 | 1 | 1 | 0 | 0 |
| CGGA1138 | 1 | 0 | 0 | 1 | 0 | 1 | 1 | 0 | 0 |
| CGGA1142 | 1 | 0 | 0 | 0 | 1 | 1 | 1 | 0 | 0 |
| CGGA1164 | 1 | 1 | 0 | 0 | 1 | 0 | 1 | 1 | 0 |
| CGGA1172 | 1 | 0 | 0 | 0 | 1 | 1 | 1 | 1 | 1 |
| CGGA1208 | 1 | 1 | 1 | 0 | 0 | 1 | 1 | 0 | 0 |
| CGGA1260 | 1 | 1 | 1 | 0 | 0 | 1 | 1 | 0 | 0 |
| CGGA1326 | 1 | 0 | 0 | 1 | 0 | 1 | 0 | 1 | 0 |
| CGGA1353 | 1 | 0 | 0 | 0 | 1 | 1 | 1 | 0 | 0 |
| CGGA1354 | 1 | 0 | 0 | 1 | 0 | 1 | 1 | 0 | 1 |
| CGGA1371 | 1 | 0 | 0 | 0 | 1 | 1 | 1 | 0 | 1 |
| CGGA1380 | 1 | 0 | 0 | 1 | 0 | 1 | 1 | 0 | 1 |
| CGGA1382 | 1 | 0 | 0 | 1 | 0 | 1 | 1 | 0 | 1 |
| CGGA1387 | 1 | 1 | 1 | 0 | 0 | 0 | 0 | 0 | 1 |
| CGGA139 | 1 | 0 | 0 | 1 | 0 | 1 | 1 | 1 | 0 |
| CGGA1391 | 1 | 0 | 0 | 1 | 0 | 1 | 1 | 0 | 0 |
| CGGA1402 | 1 | 0 | 0 | 0 | 1 | 1 | 1 | 0 | 1 |
| CGGA1403 | 1 | 0 | 0 | 1 | 0 | 1 | 1 | 0 | 1 |
| CGGA1410 | 1 | 0 | 0 | 1 | 0 | 1 | 1 | 0 | 1 |
| CGGA1419 | 1 | 1 | 0 | 1 | 0 | 1 | 1 | 0 | 0 |
| CGGA1420 | 1 | 0 | 0 | 1 | 0 | 1 | 1 | 0 | 1 |
| CGGA1426 | 1 | 0 | 1 | 0 | 0 | 1 | 1 | 0 | 0 |
| CGGA1429 | 1 | 1 | 0 | 0 | 1 | 1 | 1 | 1 | 1 |
| CGGA1430 | 1 | 1 | 1 | 0 | 0 | 1 | 1 | 0 | 0 |
| CGGA1433 | 1 | 0 | 0 | 1 | 0 | 1 | 1 | 0 | 0 |
| CGGA1441 | 1 | 0 | 0 | 0 | 1 | 1 | 1 | 0 | 1 |
| CGGA1444 | 1 | 0 | 0 | 1 | 0 | 1 | 1 | 0 | 1 |
| CGGA1451 | 1 | 0 | 0 | 1 | 0 | 1 | 1 | 0 | 0 |
| CGGA1452 | 1 | 0 | 0 | 1 | 0 | 1 | 1 | 0 | 1 |
| CGGA1457 | 1 | 0 | 0 | 1 | 0 | 1 | 1 | 0 | 1 |
| CGGA1461 | 1 | 0 | 0 | 1 | 0 | 1 | 1 | 0 | 0 |
| CGGA1462 | 1 | 0 | 1 | 0 | 0 | 1 | 1 | 0 | 1 |
| CGGA1478 | 1 | 0 | 0 | 1 | 0 | 1 | 1 | 0 | 1 |
| CGGA1480 | 1 | 1 | 0 | 1 | 0 | 1 | 1 | 0 | 1 |
| CGGA1491 | 1 | 0 | 0 | 1 | 0 | 1 | 1 | 1 | 0 |
| CGGA1492 | 1 | 1 | 0 | 1 | 0 | 0 | 0 | 0 | 0 |
| CGGA1500 | 1 | 0 | 1 | 0 | 0 | 1 | 1 | 0 | 0 |
| CGGA1505 | 1 | 1 | 1 | 0 | 0 | 1 | 1 | 1 | 0 |
| CGGA1507 | 1 | 1 | 0 | 1 | 0 | 1 | 1 | 0 | 0 |
| CGGA1521 | 1 | 0 | 0 | 1 | 0 | 1 | 1 | 0 | 1 |
| CGGA1537 | 1 | 0 | 1 | 0 | 0 | 0 | 0 | 0 | 1 |
| CGGA1538 | 1 | 1 | 0 | 1 | 0 | 1 | 1 | 0 | 1 |
| CGGA1539 | 1 | 0 | 0 | 0 | 1 | 1 | 1 | 1 | 1 |
| CGGA1541 | 1 | 1 | 1 | 0 | 0 | 1 | 1 | 0 | 1 |
| CGGA1543 | 1 | 0 | 0 | 1 | 0 | 1 | 0 | 1 | 0 |
| CGGA1546 | 1 | 0 | 0 | 1 | 0 | 0 | 1 | 0 | 0 |
| CGGA1548 | 1 | 0 | 0 | 0 | 1 | 1 | 1 | 0 | 0 |
| CGGA1558 | 1 | 1 | 1 | 0 | 0 | 1 | 1 | 0 | 1 |
| CGGA1559 | 1 | 0 | 0 | 1 | 0 | 0 | 1 | 1 | 1 |
| CGGA1560 | 1 | 0 | 0 | 1 | 0 | 1 | 1 | 1 | 1 |
| CGGA1564 | 1 | 0 | 1 | 0 | 0 | 1 | 1 | 0 | 0 |
| CGGA1571 | 1 | 0 | 0 | 1 | 0 | 1 | 1 | 1 | 1 |
| CGGA1586 | 1 | 0 | 0 | 1 | 0 | 1 | 1 | 0 | 0 |
| CGGA1596 | 1 | 0 | 0 | 1 | 0 | 1 | 1 | 0 | 1 |
| CGGA1597 | 1 | 0 | 1 | 0 | 0 | 1 | 0 | 0 | 1 |
| N489 | 0 | 0 | 0 | 0 | 1 | 0 | 0 | 0 | 0 |
| N491 | 0 | 0 | 0 | 0 | 1 | 0 | 0 | 0 | 0 |
| N494 | 0 | 0 | 0 | 0 | 1 | 0 | 0 | 0 | 0 |
| N499 | 0 | 0 | 0 | 0 | 1 | 0 | 0 | 0 | 0 |
| N505 | 0 | 0 | 0 | 0 | 1 | 0 | 0 | 0 | 0 |
| N508 | 0 | 0 | 0 | 0 | 1 | 0 | 0 | 0 | 0 |
| N509 | 0 | 0 | 0 | 0 | 1 | 0 | 0 | 0 | 0 |
| N512 | 0 | 0 | 0 | 0 | 1 | 0 | 0 | 0 | 0 |
| N530 | 0 | 0 | 0 | 0 | 1 | 0 | 0 | 0 | 0 |
| N548 | 0 | 0 | 0 | 0 | 1 | 0 | 0 | 0 | 0 |
| N557 | 0 | 0 | 0 | 0 | 1 | 0 | 0 | 0 | 0 |
| N562 | 0 | 0 | 0 | 0 | 1 | 0 | 0 | 0 | 0 |
| N573 | 0 | 0 | 0 | 0 | 1 | 0 | 0 | 0 | 0 |
| N577 | 0 | 0 | 0 | 0 | 1 | 0 | 0 | 0 | 0 |
| N588 | 0 | 0 | 0 | 0 | 1 | 0 | 0 | 0 | 0 |
| N593 | 0 | 0 | 0 | 0 | 1 | 0 | 0 | 0 | 0 |
| N597 | 0 | 0 | 0 | 0 | 1 | 0 | 0 | 0 | 0 |
| N609 | 0 | 0 | 0 | 0 | 1 | 0 | 0 | 0 | 0 |
| N775 | 0 | 0 | 0 | 0 | 1 | 0 | 0 | 0 | 0 |
| N877 | 0 | 0 | 0 | 0 | 1 | 0 | 0 | 0 | 0 |

## Table S3: Detail information of Drug compounds.

| Articles | Drugs |
| --- | --- |
| Investigational New Drugs for Brain Cancer | Procarbazine, CCNU/Lomustine, Vincristine, temozolomide, bevacizumab , erlotinib, gefitinib, lapatinib, cetuximab, pazopanib, Dacomitinib, Afatinib, cetuximab, panitumumab, nimotuzumab, AMG 595, Sym004, ABT-414, imatinib mesylate, sunitinib, sorafenib, pazopanib, dasatinib, crenolanib, cabozanitinib, rilotumumab, onartuzumab, cediranib, pazopanib, vandetanib, vatalanib, everolimus, sapanisertib, buparlisib, Oncothyreon, XL147, Vemurafenib, dabrafenib, trametinib, selumetinib, decitabine, 5-azacytidine, Dichloroacetate, Gabapentin, vorinostat, valproic acid, panobinostat, Rindopepimut, HSPPC-96, ICT-107, nivolumab, pidilizumab, pembrolizumab, ipilimumab, pidilizumab, |
| Novel chemotherapeutic agents for the treatment of brain cancer | Temozolomide, marimastat, batimastat, AG3340, BAY 12-9566, Irinotecan, angiostatin, endostatin, suramin, thalidomide, TNP-470, marimistat, SU-6668, retinoids, paclitaxel, BCNU, |

## Table S4: Oncodriver genes with their corresponding modules.

| **Gene** | **module** |
| --- | --- |
| MUC16 | grey |
| WDR89 | grey |
| ANKRD36 | greenyellow |
| MTCH2 | grey |
| SSC5D | grey |
| FLG | blue |
| TMEM163 | grey |
| KMT2C | greenyellow |
| GNAQ | blue |
| IRS2 | grey |
| DLX6 | grey |
| VWF | grey |
| GXYLT1 | greenyellow |

## Table S5**:** Detail information of proteins used in docking analysis.

| Protein name | Uniprot_id | Template ID |
| --- | --- | --- |
| MTCH2 | Q9Y6C9 | AF-Q9Y6C9-F1 |
| VWF | P04275 | 3GXB |
| WDR89 | Q96FK6 | AF-Q96FK6-F1 |

## Table S6**:** Protein change information of the oncodriver genes (Proteins)

| Gene name | Position | Counts | Variant_Classification | **Effect** | |
| --- | --- | --- | --- | --- | --- |
|  |  |  |  | PolyPhen-2 | SNPs&GO |
| MTCH2 | R77* | 2 | Nonsense_Mutation | - | - |
|  | C79H | 2 | Missense_Mutation | Probably damaging | Disease |
|  | F237S | 4 | Missense_Mutation | - | Disease |
|  | V246A | 3 | Missense_Mutation | Probably damaging | Disease |
| VWF | R1342C | 1 | Missense_Mutation | Probably damaging | Disease |
|  | L1503P | 2 | Missense_Mutation | Probably damaging | Disease |
|  | S1506L | 2 | Missense_Mutation | Probably damaging | Disease |
| WDR89 | K57R | 3 | Missense_Mutation | Benign | - |
|  | R64* | 2 | Nonsense_Mutation | - | - |
|  | P70S | 1 | Missense_Mutation | Possibly damaging | Neutral |
|  | R100* | 1 | Nonsense_Mutation | - | - |
|  | R103S | 4 | Missense_Mutation | Benign | Neutral |
|  | G112D | 1 | Missense_Mutation | Probably damaging | Neutral |
|  |  |  |  |  |  |

# Supplementary Figures

**
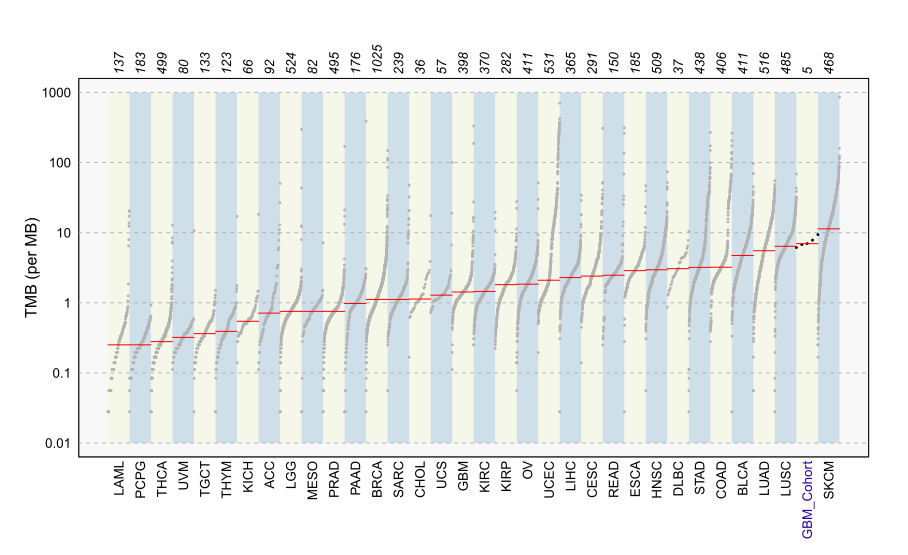
**

**Fig.S1** Comparison of Tumour mutational burden (TMB) found in our GBM cohort (blue colour) against 33 TCGA cohorts. The red horizontal lines show the median number of mutations in each of the corresponding cancer types, and vertical lines show the number of mutation per mega base, where each dot represents a sample. The number of samples are represented in top.


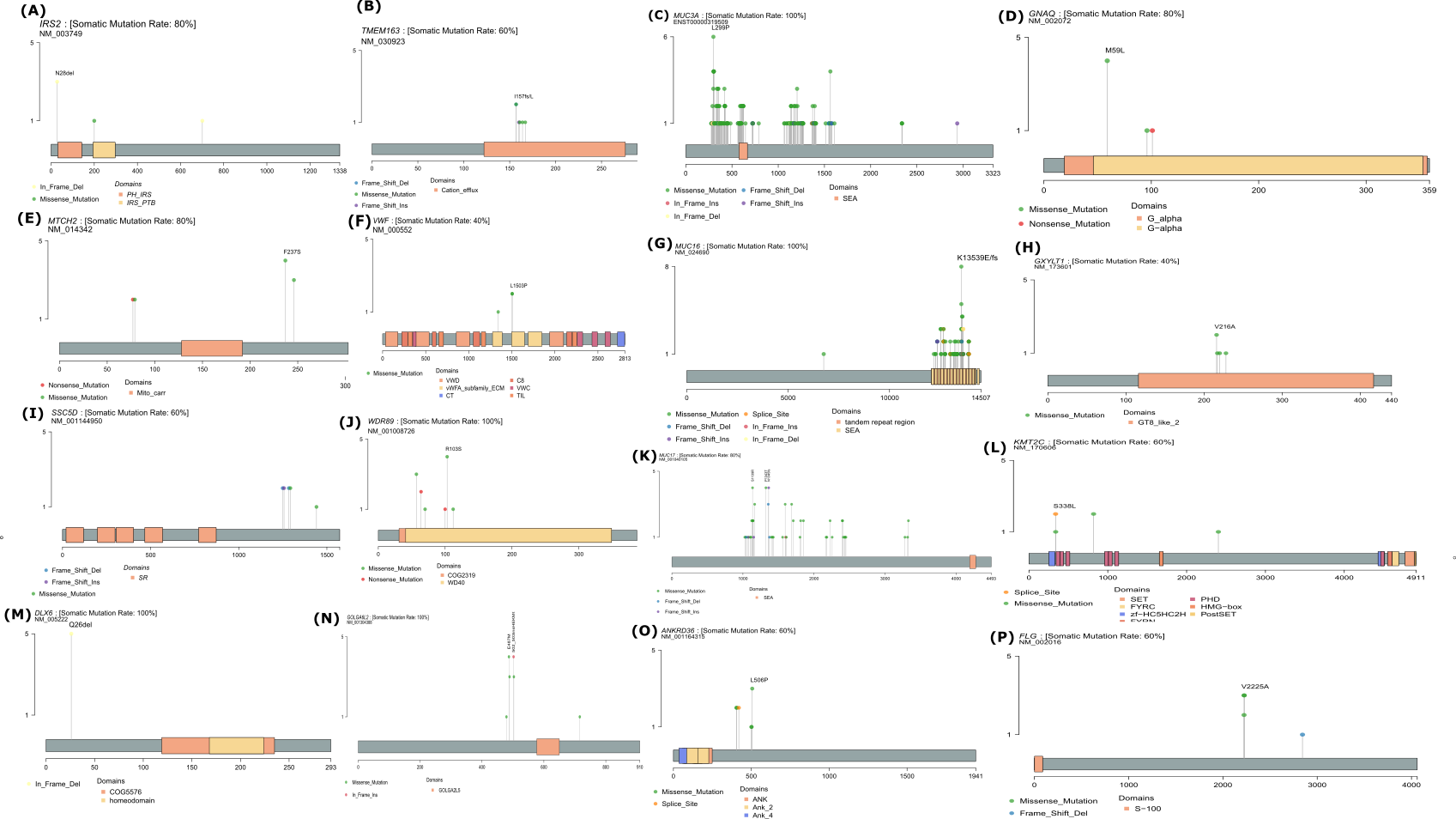


**Fig.S2:** **(A-P)** Lollipop plot to visualize where mutations occur within the oncodriver genes (proteins), highlighting their domains, recurrent hotspots.


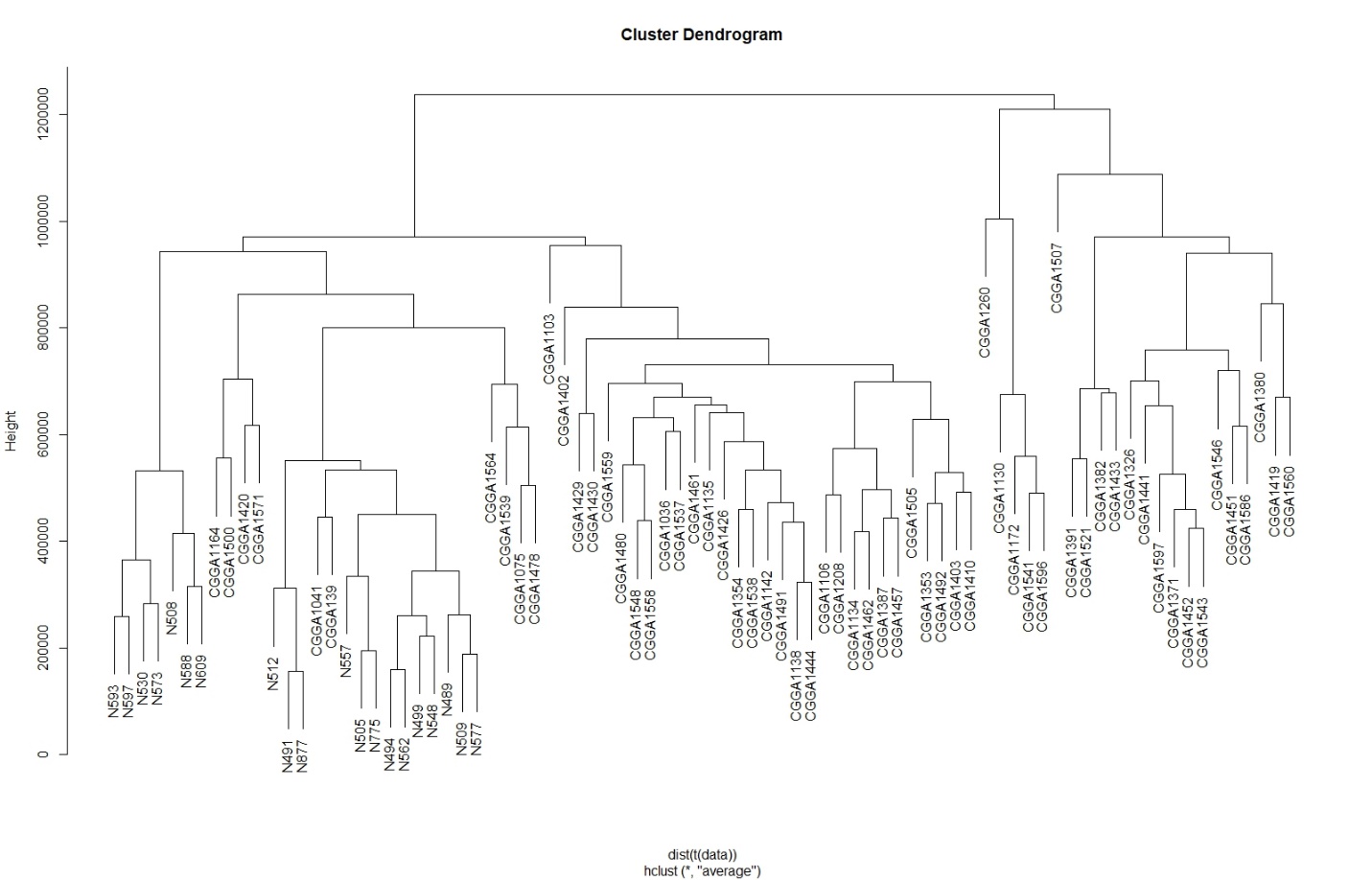


**Fig.S3:** Hierarchical clustering to detect outlier.
